# Crowd cluster data in the USA for analysis of human response to COVID-19 events and policies

**DOI:** 10.1038/s41597-023-02176-1

**Published:** 2023-05-10

**Authors:** B. Swaminathan, J. Kang, K. Vaidya, A. Srinivasan, P. Kumar, S. Byna, D. Barbarash

**Affiliations:** 1 University of California, Berkeley, USA; 2 University of West Florida, Pensacola, USA; 3 Florida State University, Tallahassee, USA; 4 Lawrence Berkeley National Lab, Berkeley, USA; 5 Purdue University, West Lafayette, USA

**Keywords:** Computer science, Risk factors

## Abstract

We provide data on daily social contact intensity of clusters of people at different types of Points of Interest (POI) by zip code in Florida and California. This data is obtained by aggregating fine-scaled details of interactions of people at the spatial resolution of 10 m, which is then normalized as a social contact index. We also provide the distribution of cluster sizes and average time spent in a cluster by POI type. This data will help researchers perform fine-scaled, privacy-preserving analysis of human interaction patterns to understand the drivers of the COVID-19 epidemic spread and mitigation. Current mobility datasets either provide coarse-level metrics of social distancing, such as radius of gyration at the county or province level, or traffic at a finer scale, neither of which is a direct measure of contacts between people. We use anonymized, de-identified, and privacy-enhanced location-based services (LBS) data from opted-in cell phone apps, suitably reweighted to correct for geographic heterogeneities, and identify clusters of people at non-sensitive public areas to estimate fine-scaled contacts.

## Background & Summary

The COVID-19 outbreak started impacting the United States (USA) in March 2020, and the USA soon became the nation with the largest number of COVID-19 cases. There was a resurgence of COVID-19 toward the end of 2020, and it is believed that increased gatherings of people indoors during cold weather has been a driver of its spread. There are wide geographic and demographic disparities on morbidity, impacted by differences in social distancing. Some of these differences arise from differences in local public health interventions and messaging, variations in the type of work and need for work, lockdown fatigue, vulnerabilities of underserved populations, etc. Researchers find a need to understand the impact of policies and events on social contacts of people at a sufficiently fine spatial resolution to account for demographic and geographic heterogeneities. In addition, analysis of fine-scaled social contact patterns can provide insight into the drivers of epidemic risk.

Conventional data sources for understanding social-contact patterns, such as airport traffic data, have low spatial granularity and lack real-time information. Consequently, researchers have resorted to the use of novel data sources, such as location-based services (LBS) data collected from smart phone apps^1,2^. Vendors sell or donate such data for research purposes, though the price for commercially sold data is a limitation for widespread research use. In addition, the analysis of tens of terabytes of data is also a computational bottleneck. Some aggregated datasets are available to the public, including the Google Community Mobility Reports (https://www.google.com/covid19/mobility) and Cuebiq’s COVID-19 Mobility Index (CMI) (https://help.cuebiq.com/hc/en-us/articles/360041285051-Mobility-Insights-Mobility-Index-CMI-). However, these typically provide coarse scale measures of traffic, such as the radius of gyration at the county level, or total traffic (number of visitors) at some specific points of interest (POI)^2,3^. Moreover, these do not directly measure social proximity. For example, a location with a large area may have few social contacts even if the traffic is high. In addition, POIs with low traffic densities may have high interactions. For example, we have observed high interactions at airport security check areas even when airport traffic was low.

Related works that use traffic or colocation^3^ at a POI as a metric for contacts do not account for such heterogeneity in population density within a POI. *Our contribution lies in accounting for these finer-scaled variations and also correcting for geographic biases in the raw mobility data*, as explained below. We later show that these geographic biases reflect demographic biases to some extent. Consequently, correcting for these is essential for modeling social trends accurately.

We identify fine-scaled clusters of crowds on each date. We then estimate the number of social contacts and their duration to yield people-minutes of social contacts. For each zip code in Florida and California, we aggregate these results by POI types and provide a normalized estimate of people-minutes of social contacts, called the social contact index, for 15 POI types. We also provide the distribution of the cluster sizes and average time spent by a person in a cluster in each POI type. Our results account for biases in the number of users at different geographic locations, in contrast to currently available datasets for the traffic. Researchers can use our social contact index and cluster characterization for a variety of purposes that need social interaction patterns of people. For example, they can analyze temporal trends in social interactions at different types of businesses^1^ and relate them to public health interventions or messaging, or use them to model infection outbreaks at events. Such analysis can also suggest interventions at specific locations that can reduce social interactions, such as changes in queue design at airport security gates.

We note that mobility data is often used for determining the *flux* between different origin-destination pairs. This has wide applications, such as in models that simulate the spread of the epidemic at a large scale. Mobility data can also be useful for determining *positions* of people. This can be useful, for example, in applications that need to know the locations and extents of social contacts, such as those described above. Our focus is on the latter class of applications, where mobility data provides *positions* of individuals. In addition, we note that our data is meant to provide a statistically unbiased measure of social contacts rather than to capture any specific event precisely. The choices made in our Methods section reflect this goal. However, not all sources of bias could be eliminated. The Usage Notes section mentions these to enable proper use of the data.

## Methods

To make anonymized, de-identified, and privacy-enhanced LBS data from opted-in cell phone apps useful for the broader research community in analyzing mobility data, we perform a thorough cleaning of the data and provide useful data on metrics of social contacts, along with metadata. We take the first steps towards making this data compliant with FAIR (Findable, Accessible, Interpretable, and Reusable) principles^4^. In achieving this goal, we start with providing extensive metadata for the data clusters, assigning a digital object identifier (DOI 10.17605/OSF.IO/YG7VZ), and publishing the data for open access on the Open Science Foundation (OSF) portal (https://osf.io/yg7vz/). We use an easy-to-use CSV file format that provides machine-readable metadata and data that can be accessed by a wide variety of programming languages including Python. In the remainder of this section, we will describe data cleaning and clustering methods and explain the metadata.

### Data source and data cleaning

Our granular privacy-enhanced data consists of LBS data for all of the USA from the vendor, Cuebiq, under their *Data For Good* program. Cuebiq obtains anonymized de-identified data using around 200 smartphone apps from users who explicitly opt in to share their data for research purposes. The vendor is compliant with the General Data Protection Regulation (GDPR) and California Consumer Privacy Act (CCPA). Most of this data has accuracy of around 10 m. It covers around 20 million adult users from all of the USA. Data is collected 120 times a day per user, which is roughly every 12 minutes. The vendor provides updates every 24 hours, so that the data is current.

The data consists of a number of records, with each record providing information on one anonymized user at one time point. A record contains the following fields: (i) anonymized de-identified user ID, (ii) timestamp, (iii) latitude of the user, (iv) longitude of the user, (v) accuracy of the record, and (v) operating system used. In order to further provide privacy, Cuebiq removes all data points from sensitive points of interest, and obfuscates users’ residential areas by transforming the true home locations to the centroid of the corresponding census block group, thereby enabling demographic inference while preserving privacy.

We removed records that are clearly incorrect. In particular, latitudes and longitudes which do not fall in the USA are removed. Records for a certain date may occasionally appear after a delay, and so are present in a dataset for a future date. We identify these based on their timestamp and the time zone for that location, which is obtained from the ZoneDetect time zone detection code (https://github.com/BertoldVdb/ZoneDetect). Around 99.5% of the records in the USA appear either along with the dataset for the correct date or the next date. We use these records, but ignore records that appear later than that. Our initial datasets covered January 1, 2020 to July 15, 2020. We currently provide clusters of crowds with data from January 1, 2020 to July 14, 2020, to account for the one extra date needed to account for the above time lag. The vendor provided a six-month dataset for this analysis as part of its Data for Good program.

We note that related works that compute a mobility network^2^ typically rely on identifying stops of some significant duration, rather than considering all records. In such applications, transient locations lack value and thus this is a reasonable solution. Our goal is to enable understanding of fine-scaled contacts. Transient presence at a location can add to infection risk between susceptible and infective individuals both from theoretical considerations in dose-response models and based on observations during the COVID-19 epidemic. For example, dose-response models for infection risk are based on total exposure in some time span^5^. In practice too, several short exposures of under a minute with cumulative time a little over 15 minutes has led to secondary COVID-19 infection^6^. Thus, including transient exposure in social contacts is desirable. We, therefore, do not remove records that do not correspond to stops.

### Clustering

**Fig. 1:**
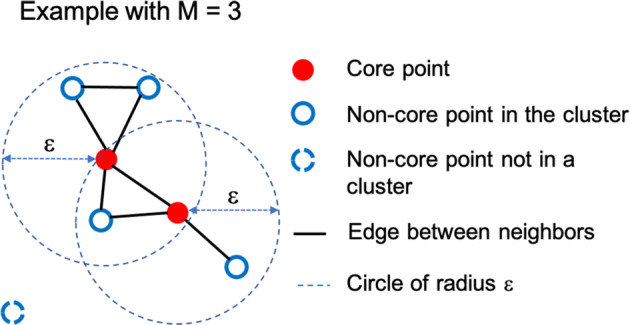
Illustration of a neighborhood graph with one component. M = 3 for ease of visualization.

We first partition the LBS data geographically for the sake of computational efficiency, first by county and then by the Census Block Group (CBG) within each county. We then use DBSCAN for clustering the records^7^ (https://scikit-learn.org/stable/modules/generated/sklearn.cluster.DBSCAN.html). DBSCAN is a popular density-based clustering algorithm that links records into the same cluster if they satisfy certain proximity conditions as given in Algorithm 1 and Fig. 1 below. For the purpose of clustering, we define points by their two spatial coordinates and timestamp. We define the ε parameter so that a point within a spatial distance of 15 m and a timestamp difference of 20 minutes would be considered to be in the ε-neighborhood. We use these parameter values because the spatial resolution of our data is around 10 m and records are generated, on average, every 12 minutes. We later show that the social contact index is not very sensitive to the specific choice of parameter values. We choose the parameter M (‘min_points’ in DBSCAN terminology) as 10 so that records are considered as core points only if a reasonable number of others appear in the vicinity .

**Table 1:** Pearson correlation coefficient between CBG population per user in January 2020 and various demographic characteristics.

The fraction of the population with the demographic characteristic (except for median income and CBG population)	Correlation with CBG population per user
African Americans	0.24
American Indians	0.09
Asian	0.11
Hawaiian	0.03
Other	0.27
White	−0.36
12th grade	0.11
GED	−0.03
High school diploma	−0.09
Bachelor’s degree	−0.05
Master’s degree	0.02
Professional degree	0.06
Male	0.02
Income less than $10,000	0.21
Income greater than $200,000	−0.01
Median income	−0.12
Median age	−0.23
CBG population	−0.04

A positive correlation suggests that the demographic group may be underrepresented in the user base.

#### Algorithm 1

Clustering for the (latitude, longitude, time) 3-tuples using DBSCAN^7^.

1. Find all points that have M other records in their ε-neighborhood.
  - Call these *core points*.
  - Any point in the ε-neighborhood of a point is considered a *neighbor* of that point.
2. Find the connected components in the neighbor graph of the core points. (Edges exist in this graph between core points that are neighbors.)
  - Label each connected component as a distinct cluster.
  - Assign each non-core point to a cluster in whose ε-neighborhood it is. If there is no such cluster, then consider the point as noise and ignore it.

### Accounting for heterogeneity

**Fig. 2:**
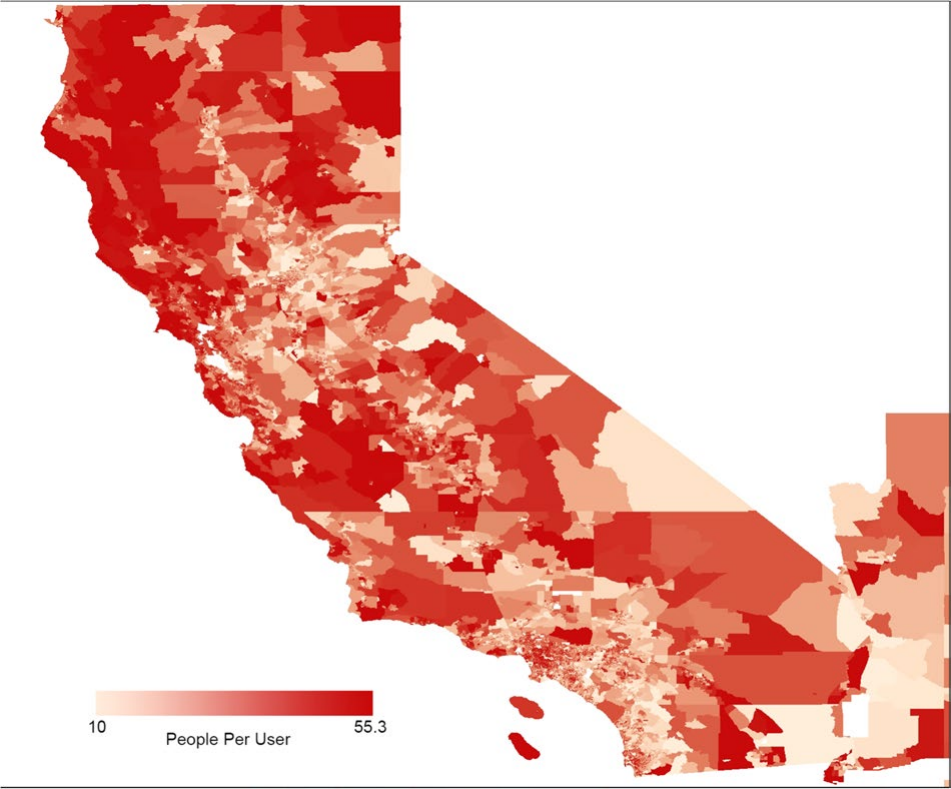
Population per user in January 2020 in California CBGs. Darker regions are underrepresented in the user base.

Once we identify the clusters, we need to estimate the number of real persons in those clusters. There are often demographic and geographic differences in the representation of people in our user base. For example, Fig. 2 shows geographic differences in the representation of individuals in our dataset. This underrepresentation is correlated with the fraction of certain minority populations in the CBGs, as shown in Table 1. It is important to correct for this so that such typically underserved populations are not underrepresented in our analysis. We correct for this using Population Size Weighting^8^ in Algorithm 2 Step 1b.

**Table 2:** Pearson correlation coefficient between the social contact index computed using (ε = 15 m, C = 15 m) and other choices of these parameters.

Spatial ε	C	Correlation coefficient	p-value
10 m	5 m	0.996	<10^−10^
15 m	10 m	>0.999	<10^−10^
15 m	20 m	>0.999	<10^−10^
20 m	25 m	0.999	<10^−10^
25 m	30 m	0.998	<10^−10^

The p-values are for the hypothesis that the results are uncorrelated.

We account for such heterogeneity by computing the number of real persons each user represents. We first estimate the home CBG for each user as follows. For each month, we consider the different CBGs in which a user spent weekend nights after 11 pm and before 9 am (based on the local time at the geolocation of each respective record) and choose the most frequent one as the user’s home CBG that month. Around 99% of users have records from only one CBG at those times, and so the home CBG is unambiguous for most users. We use this data to determine the number of users from each CBG. We then use the American Community Survey data (https://www.census.gov/programs-surveys/acs) for the population of each CBG to estimate the number of persons represented by each user. If this number is high for a CBG, then there are not many users in that CBG relative to its population.

### Social contact index

In each cluster, we determine the unique users and use these to estimate the number of real persons there from the above result. In this process, if the number of persons each user represents is below the 5^th^ percentile or above the 95^th^ percentile based on the calculation above, then we replace these with the 5^th^ and 95^th^ percentile, respectively, so that outliers would not affect the estimate. This results in each user representing between roughly 10 and 55 real persons. We compute the number of records in the cluster in the time frame of that cluster and combine it with the number of unique users to estimate the time spent by each user. Combining it with the areas, we estimate the number of contacts per unit time, and hence the people-minutes of contact, as shown in Algorithm 2. We then divide this by the total number of social contacts in the USA on Jan 8 and multiply by a scaling factor of 1,000,000 to give the social contact index for each cluster.

#### Algorithm 2

Computing Social Contacts for each cluster.

1. N ← Estimated number of real persons in the cluster, computed as follows.
  - Determine each unique user in the cluster.
  - Sum the number of people each user represents, based on the number of people per user in each user’s home CBG. This sum is the value of N.
2. A ← Area of cluster determined by the smallest bounding box that includes all the points in the cluster.
3. T ← Cluster duration, determined by difference in timestamps of the latest and earliest records in the cluster.
4. N_C_ ← Number of distinct circles in which records could lie computed as ceil[A/(πC^2^)], where C is the contact threshold, taken as 15 m.
5. T_u_ ← Estimated average time spent by a user in that cluster = number of records in the cluster/(number of unique users in the cluster x average number of records per user per unit time over that CBG that date).
6. N_P_ ← Estimated number of persons in each circle = ceil[N/N_C_)].
7. S ← Number of person-minutes of social contacts in the cluster = NT_u_(N_P_ - 1). A cluster is ignored if this value is not greater than 0.

Algorithm 2 estimates the number of contacts based on the area of the cluster, its duration, number of persons in the area, and their average time in the cluster. It is best understood by first considering an asymptotic situation with a large number of persons uniformly distributed in a large area. N/A gives the number of persons per unit area. NπC^2^/A gives the number of persons, N_P_, within the contact threshold of any person, including that person. N_P_-1 gives the number of contacts for that person. When multiplied by the average residence time T_u_, we get the people-minutes of contact per person. When multiplied by, N, we get the total number of contact minutes as NT_u_(N_P_ - 1) with N_P_ = NπC^2^/A. The ceilings mentioned in Algorithm 2 are insignificant in the asymptotic situation. Steps 4, 6, and 7 give the same expression for contacts as above. This justifies Algorithm 2 in the asymptotic situation.

There are two additional changes with respect to the above description. The first is that the average time is estimated in Step 5 from the number of records in the cluster, the average number of records per unit time for a user in that CBG, and the number of unique users in the cluster. The expression for this estimate is straightforward, but the rationale for using this, rather than directly estimating the dwell time of each user in a cluster, may not be apparent. The reason for our choice is that our clusters are often fine-grained spatially and temporally. Consequently, users often do not spend enough time in the cluster to estimate their dwell time directly. The averaging procedure used here avoids this problem. We provide one additional detail on the computation of the average number of records per unit time. The number of records per unit time is often low during sleeping hours, and so the average number of records per unit time would be higher during waking hours than a simple average would indicate. Instead, we compute the average number of records for each hour of the day and take the weighted average over hours in which records are present, with the weights being the number of records in each hour.

The second aspect of Algorithm 2 is the use of ceilings and the introduction of the idea of the number of distinct circles. This does not have an impact on the asymptotic case. The ceilings used in the computation of N_C_ and N_P_ are used to account for situations with few people or small areas. In this case, people could stay farther away from each other than a uniform distribution would imply. For instance, two persons could be at opposite corners of a room. So, these persons could be assigned to distinct non-intersecting circles of radius C even if a uniform distribution would place them closer. The idea here is that due to the quadratic nature of interactions of people in close proximity, it is preferable for people to have multiple smaller groups of people than fewer larger groups of people. In providing a metric for contacts, we assume that people would try to separate into such smaller groups in such situations. So, we try to separate them into as many distinct circles as possible, with each circle having radius of the contact threshold.

### Aggregation

We add the social contact index for all clusters in a POI in our database to compute the social contact index for that POI. If the cluster does not fall entirely within that POI, then we multiply the contact index by the fraction of the cluster’s area that falls within that POI. The list of POIs was a freely available list from Safegraph, and the list from 2021, used by us, contained over 4.5 million POIs in the USA. Due to restrictions on the use of our mobility data, we chose a subset of 84 subcategories from California and Florida and classified them into fewer broad categories. These categories and subcategories are provided in our dataset.

The area of a POI is defined by a radius around its center, with different radii defined for different POI types as provided by Cuebiq. We mapped Cuebiq categories to equivalent Safegraph categories (sometimes multiple). For certain exceptional locations, such as amusement parks in Orlando, we explicitly used their locations from google maps data, because the defaults were not realistic. The POI categories, subcategories, and default radii are provided in our dataset as described below. The contacts for each POI of the same type in each zip code are added to yield the social contact index for that POI category. If A is the area of a cluster’s overlap with a POI, then A^0.5^ defines a characteristic size for that cluster. We provide parameters to characterize the distribution of sizes for each POI type. We also compute the average time spent by a person in a cluster for each POI type and the number of clusters in each POI type.

## Data Records

The datasets are available on the Open Science Framework – Center for Open Science^9^. This may be updated with datasets for new dates as they are processed. The data consist of (i) one comma-separated value (CSV) file with POI types and their radii, (ii) the main cluster data, organized in two CSV files per date, one file for each of Florida and California. Both file types are explained below.

- POI.csv. It provides the size range for each POI subcategory. A POI type may include subcategories with different radii. For example, ‘Automotive’ would include car dealers and car repair shops, which would have different radii. Therefore, we provide these details. This file has the following fields.
  - POI_Type is a string identifying the POI type.
  - POI_Subtype is a string identifying the POI subcategory.
  - Radius is the radius in meters.
  CSV files for each date are named in the format <Year> <Month> <date> in a folder named <State>, as illustrated in the following example.
- 20200102 in the folder named *Florida* is a file that contains data for Florida in January 2, 2020. It contains the following fields.
  - ZCTA5 is a 5-digit Zip Code Tabulation Area code assigned by the US Census bureau, which corresponds to a geographic region that is a good approximation to the zip code.
  - POI_Type is a string describing the type of the POI, such as airport or grocery.
  - Number_of_Clusters is the number of clusters for that POI type in that zip code.
  - Cluster_Size_Mean is the average size of a cluster for that POI type in meters.
  - Cluster_Size_Variance is variance for the distribution of sizes of clusters for that POI type.
  - Avergage_Time is the average time spent by a person in a cluster at that POI type in minutes.
  - SCI is the Social Contact Index for that POI, as defined earlier.

**Fig. 3:**
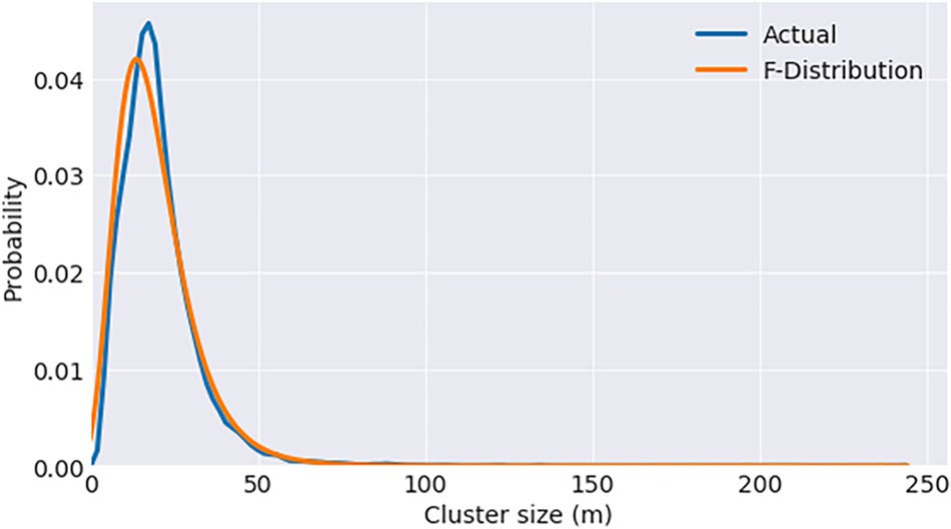
F-distribution fit to the histogram of cluster sizes in California on 1 January 2020.

Figure 3 shows the histogram of cluster sizes in California on 1 January 2020. We can see the that F-distribution fits it reasonably well. We fit such histograms for several dates for California and Florida against the F-distribution, chi-square, Weibull, lognormal, gamma, and power lognormal. The F-distribution was the only one that fit all cases reasonably. Users could use the mean and variance to compute the F-statistic parameters if they needed to.

## Technical Validation

### Sensitivity analysis of the social contact index

We perform a sensitivity analysis of the social contact index with respect to the DBSCAN parameters. Table 2 shows that the temporal trends in the social contact index for Florida, obtained by summing all the clusters in Florida, are similar with different choices of parameter values.

### Comparison with alternative data sources

Alternative data sources provide traffic information (number of users or people in a POI), which is non-linearly related to contacts. Consequently, we perform two types of comparisons. First, we compare the traffic (number of people) implied by our input LBS data with traffic from other sources, and compare them using the Pearson correlation coefficient. We then compare traffic from alternate data sources against the social contact index. The relationship between traffic and contacts can be expected to be non-linear, and so we use the Spearman correlation coefficient for such analysis. We also give the p-value for the hypothesis that the data sets are uncorrelated. We can see below that the p-values are very low, indicating that the hypothesis would be rejected for any reasonable value of significance.

We first compare our estimate of traffic (number of people) at certain types of businesses in Orlando, Florida against Safegraph (https://www.safegraph.com/covid-19-data-consortium) data (number of people) for the same businesses in Table 3. This traffic data uses the actual shapes of the businesses from Safegraph’s dataset, rather than estimating area from a standard radius for the POI type. In these tables, we have shown results for specific subcategories, in order to demonstrate that the data is accurate even without the coarser aggregation for POI types. For example, ‘Automotive Repair and Maintenance’ is a subcategory of the ‘Automotive’ POI type.

**Table 3:** Pearson correlation coefficient between traffic from Cuebiq LBS data and Safegraph data for businesses in Orlando, Florida from March 1 to May 31, 2020.

Type of business	Pearson correlation coefficient	p-value
Restaurants and Other Eating Places	0.972	<10^−10^
Department Stores	0.980	<10^−10^
Medical care	0.954	<10^−10^
Automotive Repair and Maintenance	0.927	<10^−10^
Home Furnishing	0.853	<10^−10^

We next compare the normalized Safegraph traffic against our normalized social contact index for the same businesses in Table 4. Here, Safegraph traffic is based on Safegraph’s data while the social contact index is computed using Cuebiq’s POI list.

**Table 4:** Spearman correlation coefficient between traffic from Safegraph data and social contact index for businesses in Orlando, Florida from April 1 to May 31, 2020.

Type of business	Correlation coefficient	p-value
Restaurants	0.976	<10^−4^
Department Stores	0.976	<10^−4^
Medical Care	0.905	0.002
Automotive Repair and Maintenance	0.976	<10^−4^
Home Furnishings	0.905	0.002

**Fig. 4:**
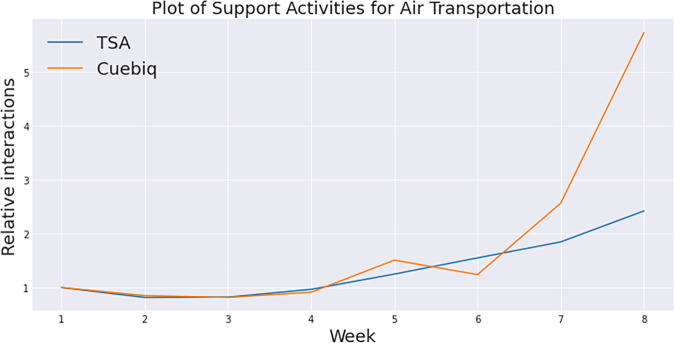
Comparison of temporal trends in USA airport traffic from TSA and social contact index at Orlando, Florida airport relative to that in Week 1. The Spearman correlation coefficient is 0.952 and the p value is 0.00026.

We finally illustrate the possibility of a non-linear relationship between traffic and contacts by comparing the relative daily airport traffic from TSA security check data (number of people) in the USA (https://www.tsa.gov/coronavirus/passenger-throughput) and the social contacts at the Orlando airport in Fig. 4. We see that the Spearman correlation is high, and that there is a non-linear relationship between traffic and contacts. We can see that days with high traffic have high contacts and those with low traffic have low contacts.

The above results show that the temporal trend in social contact index correlates well with those for alternate data sources, and also that metrics for contacts can provide better quantitative insight into interactions than traffic does.

## Usage Notes

This data can be used to understand the impact of public health interventions and messaging on social distancing. Unlike data that relies on traffic, the social contact index would give a more direct measure of distancing. Furthermore, this dataset can be used in epidemic models. These models typically need to estimate the exposure risk of people and often make assumptions like homogeneous mixing to deal with the lack of information on actual interaction patterns. Our dataset can provide such models more detailed information on contact patterns, leading to more accurate models. Recent modeling efforts for COVID-19 use LBS data to estimate exposure at different types of locations, such as restaurants, grocery, etc^1^. These are based on total traffic and also don’t account for heterogeneity in user representation. Our dataset accounts for such heterogeneity and also provides an estimate of contacts, promising more accurate data for such models.

**Fig. 5:**
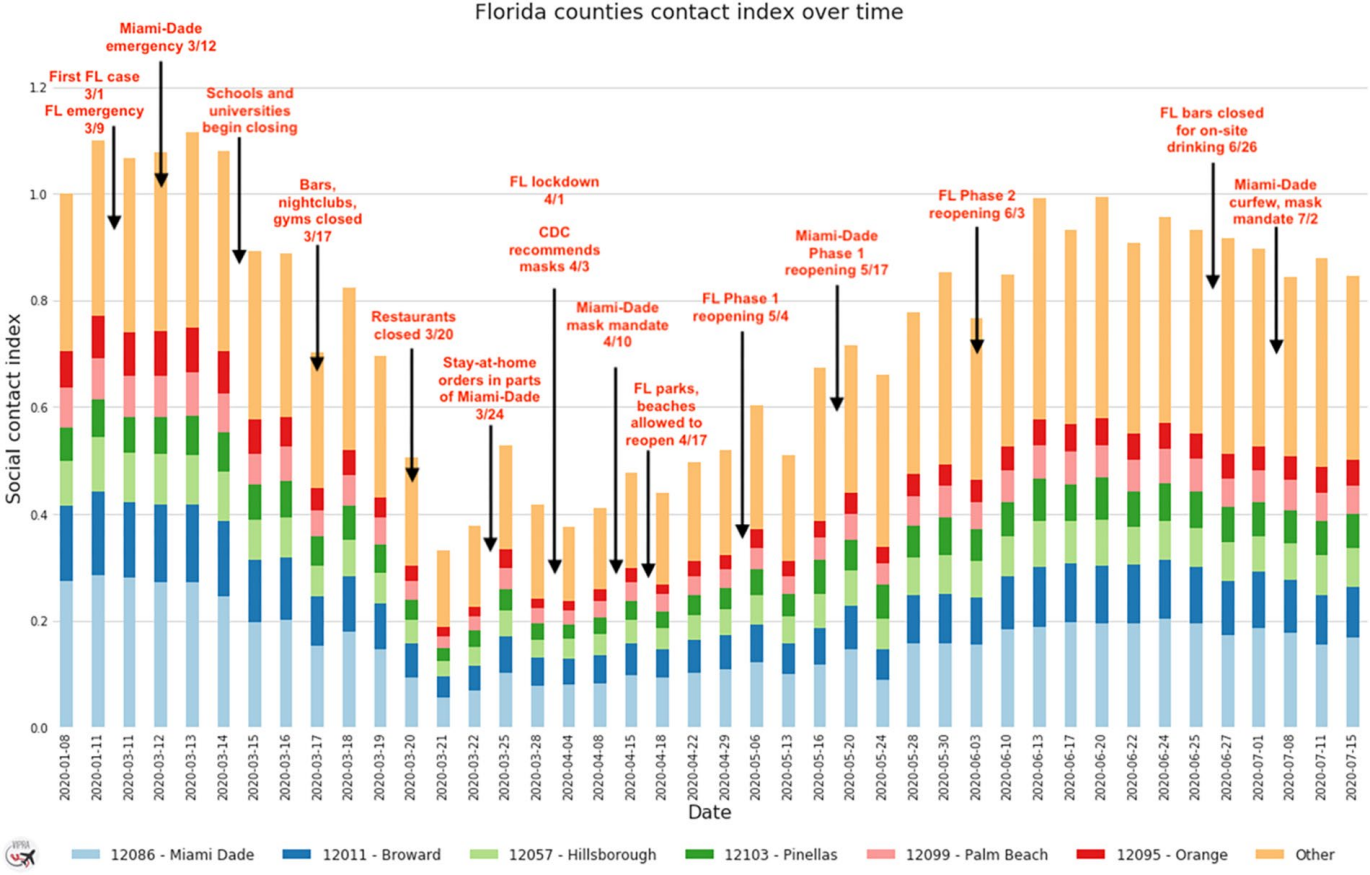
Temporal trend in social contact across Florida.

Figure 5 illustrate a type of use case for our data. It is based on the total estimated social contacts in Florida (not limited to the POIs categories), normalized against data from January 8, 2020. It suggests that social contacts decreased due to business restrictions, but that the actual lockdown did not limit social contacts. In fact, it increased after that.

This dataset has the following limitations. First, it includes only non-sensitive places. In particular, it excludes mental health facilities, homeless shelters, and jails. Consequently, crowds in such places will not be captured in our data. Second, children are not included, because all users are adults. In places frequented disproportionately by children, such as playgrounds, the dataset may underestimate social contacts. Furthermore, there are small demographic biases in the users represented in the dataset. By accounting for geographic heterogeneity at the CBG level, we address this to a reasonable extent. However, there could be some remaining bias that is not corrected by such weighting. Yet another source of bias could arise from heterogeneity in activities; persons visiting certain locations may be overrepresented in the user base. For example, our analysis in a different context suggests that people who fly are overrepresented in the user base. So, the data may be better at comparing temporal trends at certain businesses than at comparing the social contacts at different types of businesses on some particular date. Models that wish to perform such a comparison ought to estimate different weights for different business categories, perhaps using other data sources. We also note that we have estimated several quantities. While these are generally unbiased estimates, extreme, unrealistic, values are possible occasionally. Users would want to define suitable criteria for outliers in use of the data in their modeling.

## Data Availability

The codes were written in C++ and Python 3. They rely on Python DBSCAN package and on MPI (https://www.mpi-forum.org) for parallelization. The code is available from the data site^9^.
